# Effects of resistance exercise, collagen ingestion and circulating oestrogen concentration on collagen synthesis in a female athlete: A case report

**DOI:** 10.1113/EP091897

**Published:** 2024-06-20

**Authors:** Joonsung Lee, Jonathan C. Y. Tang, John Dutton, Rachel Dunn, William D. Fraser, Kevin Enright, David R. Clark, Claire E. Stewart, Robert M. Erskine

**Affiliations:** ^1^ School of Sport and Exercise Sciences Liverpool John Moors University Liverpool UK; ^2^ Bioanalytical Facility, Norwich Medical School University of East Anglia Norwich UK; ^3^ Clinical Biochemistry, Departments of Laboratory Medicine Norfolk and Norwich University Hospital NHS Foundation Trust Norwich UK; ^4^ Departments of Diabetes and Endocrinology Norfolk and Norwich University Hospital NHS Foundation Trust Norwich UK; ^5^ School of Health Sciences Robert Gordon University Aberdeen UK; ^6^ Division of Surgery and Interventional Science, Institute of Sport, Exercise and Health University College London London UK

**Keywords:** connective tissue, female, glycine, oestrogen, proline

## Abstract

We investigated the effects of resistance exercise (RE), hydrolysed collagen (HC) ingestion and circulating oestrogen concentration on collagen synthesis in a naturally menstruating female CrossFit athlete. In a double‐blind, randomised cross‐over design, the participant (36 years; height 1.61 m; mass 82.6 kg) consumed 0 or 30 g HC prior to performing back‐squat RE when endogenous circulating oestrogen concentration was low (onset of menses, OM) and high (late follicular phase, LF) during two consecutive menstrual cycles. Ten 5‐mL blood samples were collected during each of the four interventions to analyse concentrations of serum 17β‐oestradiol, and biomarkers of type I collagen turnover, that is serum procollagen type I N‐terminal propeptide (PINP, a biomarker of collagen synthesis) and plasma β‐isomerised C‐terminal telopeptide of type I collagen (β‐CTX, a biomarker of collagen breakdown), as well as the serum concentration of 18 collagen amino acids. 17β‐Oestradiol concentration was 5‐fold higher at LF (891 ± 116 pmol L^−1^) than OM (180 ± 13 pmol L^−1^). The PINP concentration × time area under the curve (AUC) was higher in the 30 g HC OM intervention (201 μg L^−1^ h) than the 30 g HC LF (144 μg L^−1^ h), 0 g HC OM (151 μg L^−1^ h) and 0 g HC LF (122 μg L^−1^ h) interventions. β‐CTX concentration decreased 1.4‐fold from pre‐RE to 6 h post‐RE in all interventions. Thus, high circulating oestrogen concentration was associated with lower collagen synthesis following RE in this female athlete. Ingesting 30 g HC, however, augmented the collagen synthesis response at LF and particularly at OM.

## Abstract (highlights)

- **What is the central question of this study?**
  Does resistance exercise‐induced collagen synthesis vary according to circulating oestrogen concentration in a naturally menstruating female athlete, and if so, does hydrolysed collagen ingestion have any impact?
- **What is the main finding and its importance?**
  Exercise‐induced collagen synthesis was low when circulating oestrogen concentration was high and vice versa. However, ingesting 30 g hydrolysed collagen prior to exercise reduced the negative effect of oestrogen on collagen synthesis. As high circulating oestrogen has been associated with greater injury risk in females, supplementing exercise with hydrolysed collagen may help protect these tissues from injury.

## 1 INTRODUCTION

The menstrual cycle of a naturally menstruating woman is regulated by the production of key hormones, of which oestrogen is one. Serum oestrogen concentration is typically low during menses, and gradually increases during the late follicular phase (LF) to its peak just before ovulation, after which it decreases during the luteal phase, then increases once more to roughly half its peak, before decreasing again prior to the start of the next cycle (Mishell et al., 1971). In addition to its role in the development and regulation of the female reproductive system, oestrogen has the potential to influence collagen turnover in human connective tissue, as oestrogen receptors are present in tendon (Bridgeman et al., 2010) and ligament (Liu et al., 1996). Thus, the fluctuation of circulating oestrogen concentration may affect the mechanical properties of these tissues (Hansen & Kjaer, 2014) at different stages of the menstrual cycle, thereby potentially explaining the greater joint laxity during LF than during menses (Park et al., 2009), which may be associated with increased injury risk during LF (Herzberg et al., 2017). Furthermore, it is known that an acute bout of resistance exercise (RE) increases tendon collagen synthesis in healthy young men (Miller et al., 2005), and ingesting 30 g hydrolysed collagen (HC) with RE augments whole body collagen synthesis more than 15 and 0 g HC in resistance‐trained young men (Lee et al., 2024). However, it is not known if HC ingestion can augment the collagen synthesis response to RE in young healthy females, and whether this response is influenced by circulating oestrogen concentration.

With this double‐blind designed case study, we had the opportunity to study in detail the metabolic responses to HC ingestion prior to RE in a young, female, naturally menstruating, CrossFit athlete at different stages of her menstrual cycle, that is when circulating oestrogen concentration was low and when it was high. We hypothesised that whole body collagen synthesis would be greater when RE was performed with 30 g HC ingestion at low circulating oestrogen concentration compared to when no HC was ingested, and to when serum oestrogen concentration was high (with or without HC ingestion).

## 2 METHODS

### 2.1 Ethical approval

This case report was registered at https://clinicaltrials.gov/ (identifier: NCT05932771), was approved by Liverpool John Moores University Research Ethics Committee (approval number: 18/SPS/059) and adhered to the *Declaration of Helsinki*.

### 2.2 Participant information

A healthy, young, naturally menstruating, female CrossFit athlete (age, 36 years; height, 1.61 m; mass, 82.6 kg) provided written informed consent before completing this study. The participant had 2 years’ experience of weightlifting, which she performed three to four times per week in addition to CrossFit training. The athlete had not used any hormonal contraceptive method for 2 years prior to the study and had a ‛normal’ menstrual cycle (MC), as determined by responses to the ‛low energy availability in females questionnaire’ (LEAF‐Q, score 1) (Melin et al., 2014). Furthermore, the participant did not smoke, and had not sustained a musculoskeletal injury in the 12 months preceding the study.

### 2.3 Experimental protocol

**FIGURE 1:**
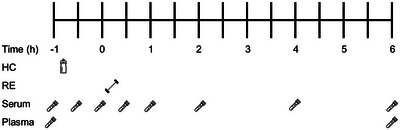
A schematic depiction of the experimental design. Hydrolysed collagen (HC) was ingested immediately after the initial blood sample, taken at −1 h. Resistance exercise (RE) was performed immediately after the blood sample taken at 0 h. Blood samples were taken at eight time points and analysed for PINP, amino acid and 17β‐oestradiol concentration (serum) and β‐CTX (plasma).

Data collection for this study began in March 2019 and was completed in May 2019. This was a double‐blind, randomised cross‐over designed case study. During the first (familiarisation) visit, the participant's barbell back squat 10‐repetition maximum (RM) load was determined, which was used as the training load during the four dose–response interventions. To ensure the muscle–tendon units bore the same mechanical loading during the four interventions, the squat depth was standardised at 90° knee flexion. Details on the exercise protocol have been reported previously (Lee et al., 2024). In addition, the duration of the participant's forthcoming MC was estimated based on self‐reporting onset of menses and previous cycle length (28 days), which was repeated for the next MC (also 28 days). Prior to the first visit to the laboratory (familiarisation session), the participant was asked the date her last period started and how many days it lasted for; and the duration of her previous MC. This information served two purposes: (i) to estimate the onset of the athlete's next menstruation and therefore the date of their first intervention, and (ii) to determine the consistency of her MC duration. Linear regression equations from the study by McIntosh et al. (1980) were used to estimate the duration of the follicular and luteal phases in the two subsequent cycles, during which the study data were collected. To ensure the greatest difference in circulating oestrogen concentration, one intervention took place at the onset of menses (OM) and one at the end of the late follicular phase (LF, interspersed by at least 12 days), and this was repeated during the subsequent MC. Thus, there were four interventions in total: OM 0 g HC; OM 30 g HC; LF 0 g HC and LF 30 g HC. Each intervention started and finished at the same times during the day (i.e., 08.00−15.00 h). The participant was instructed to consume the same evening meal (with no additional snacks) prior to the ≥10 h fast leading up to each of the four interventions. Details of the evening meal are as follows: 5% fat mince beef (125 g), chopped tomatoes (1/4 tin), Edamame pasta (50 g), ½ small onion, ½ yellow pepper and ½ cube of Kallo yeast free vegetable stock. Upon arrival at the laboratory following the overnight fast, the participant consumed either 30 or 0 g HC prior to performing four sets’ 10‐RM back squat. After completion of the exercise, the participant rested for 6 h and 10 × 5‐mL blood samples were collected over the course of the 7‐h intervention (Figure 1). Following ingestion of the supplement, only water was allowed to be consumed ad libitum during each intervention.

### 2.4 Nutritional supplementation

Thirty grams HC (Myprotein, Northwich, UK) or energy‐matched 34.1 g maltodextrin (0 g HC, Myprotein, Northwich, UK) with 50 mg vitamin C (Holland and Barrett Retail Limited, Nuneaton, UK) were dissolved with 300 mL water in an opaque drinks bottle. Although both supplements were ‘non‐flavoured’, 4 g non‐caloric sweetener (Truvia®, SilverSpoon, Peterborough, UK) was added in all drinks to mask any potential difference in taste. A laboratory technician made up the supplement, randomly assigned the different HC doses according to menstrual cycle phase and recorded the date and HC dose. Both the participant and investigators were blinded until after all measurements were analysed.

### 2.5 Blood sampling and analysis

Details of blood sampling and analyses have been reported previously (Lee et al., 2024). Briefly, all venous blood samples were collected using a catheter (22G, BD Bioscience, San Jose, CA, USA) from the right antecubital fossa. A serum sample was taken at rest immediately prior to supplement ingestion in all four interventions and was analysed for 17β‐oestradiol via enzyme‐linked immunosorbent assay (ELISA). The ELISA absorbance readings were performed at 450 nm, using a Clariostar microplate reader (BMG Labtech, Ortenberg, Germany). Six serum samples (for analysis of procollagen type 1 N‐terminal (PINP) and 18 amino acids), and two plasma samples (β‐isomerised C‐terminal telopeptide of type I collagen (β‐CTX)) were collected during each intervention (Figure 1). PINP concentration was measured by ELISA (USCN Life Sciences, China), β‐CTX concentration using electrochemiluminescence immunoassay (Roche Diagnostics, Mannheim, Germany) and 18 amino acids concentrations were measured using liquid chromatography–tandem mass spectrometry. Intra and inter‐assay coefficient of variations for these analyses were <12%. The concentration × time area under the curve (AUC) for PINP and amino acids were calculated using Prism software (version 9.4.1, GraphPad Software, Boston, MA, USA).

## 3 RESULTS

**FIGURE 2:**
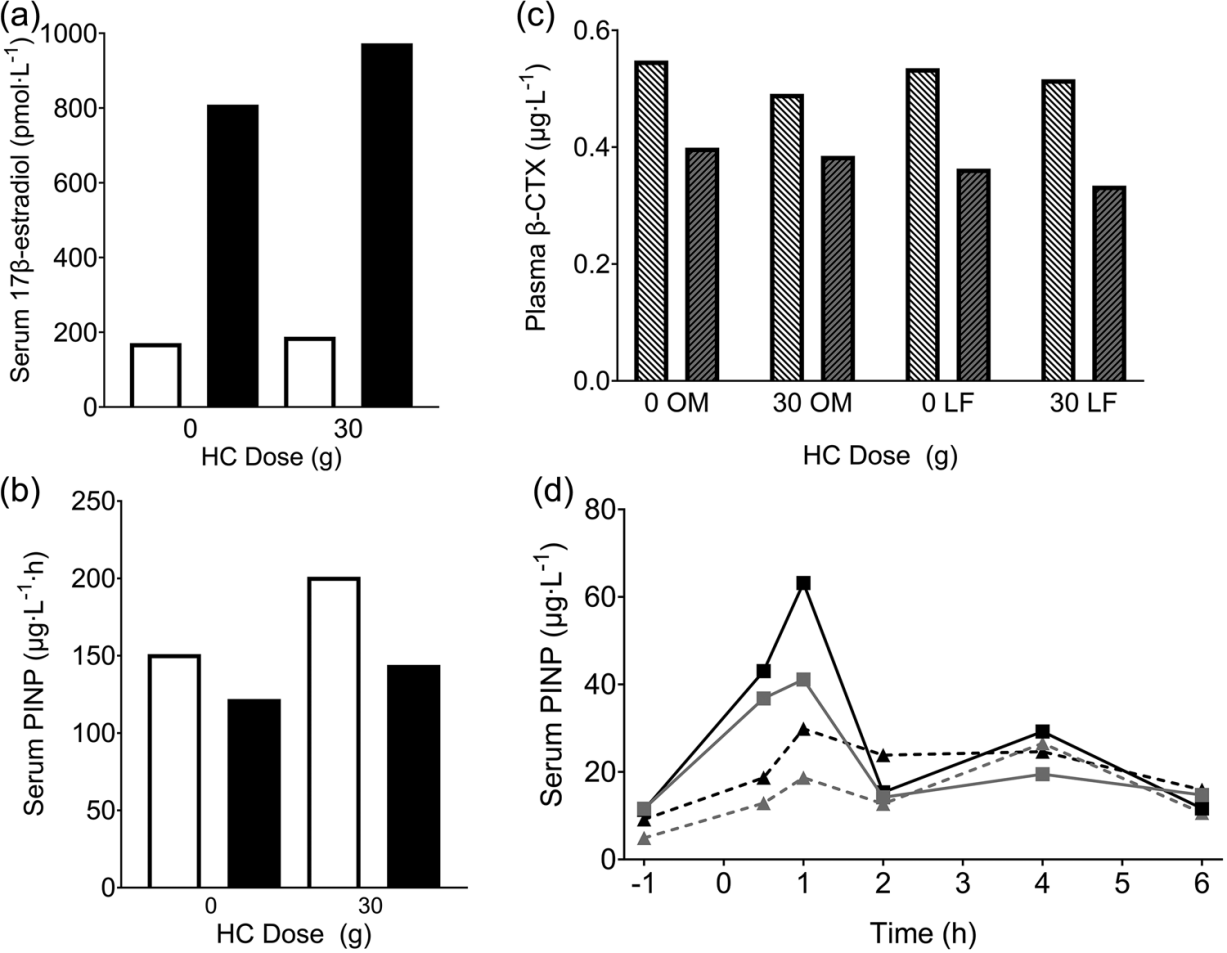
(a) Serum oestrogen concentration at rest prior to consuming 0 or 30 g hydrolysed collagen (HC) at the onset of menses (OM, white bars) and at the end of the late follicular phase (LF, black bars). (b) Serum PINP concentration × time area under the curve at OM (white bars) and LF (black bars) following either 0 or 30 g HC consumption and resistance exercise (RE). (c) Plasma β‐CTX concentrations at rest prior to either 0 or 30 g HC consumption (white bars, black‐striped) and 6 h‐post RE (grey bars, black‐striped) at OM and LF. (d) Time course of serum PINP concentration following 30 g HC consumption and RE at the OM (black squares), 0 g HC at OM (grey squares), 30 g HC at the LF (black triangles) and 0 g HC at LF (grey triangles).

**FIGURE 3:**
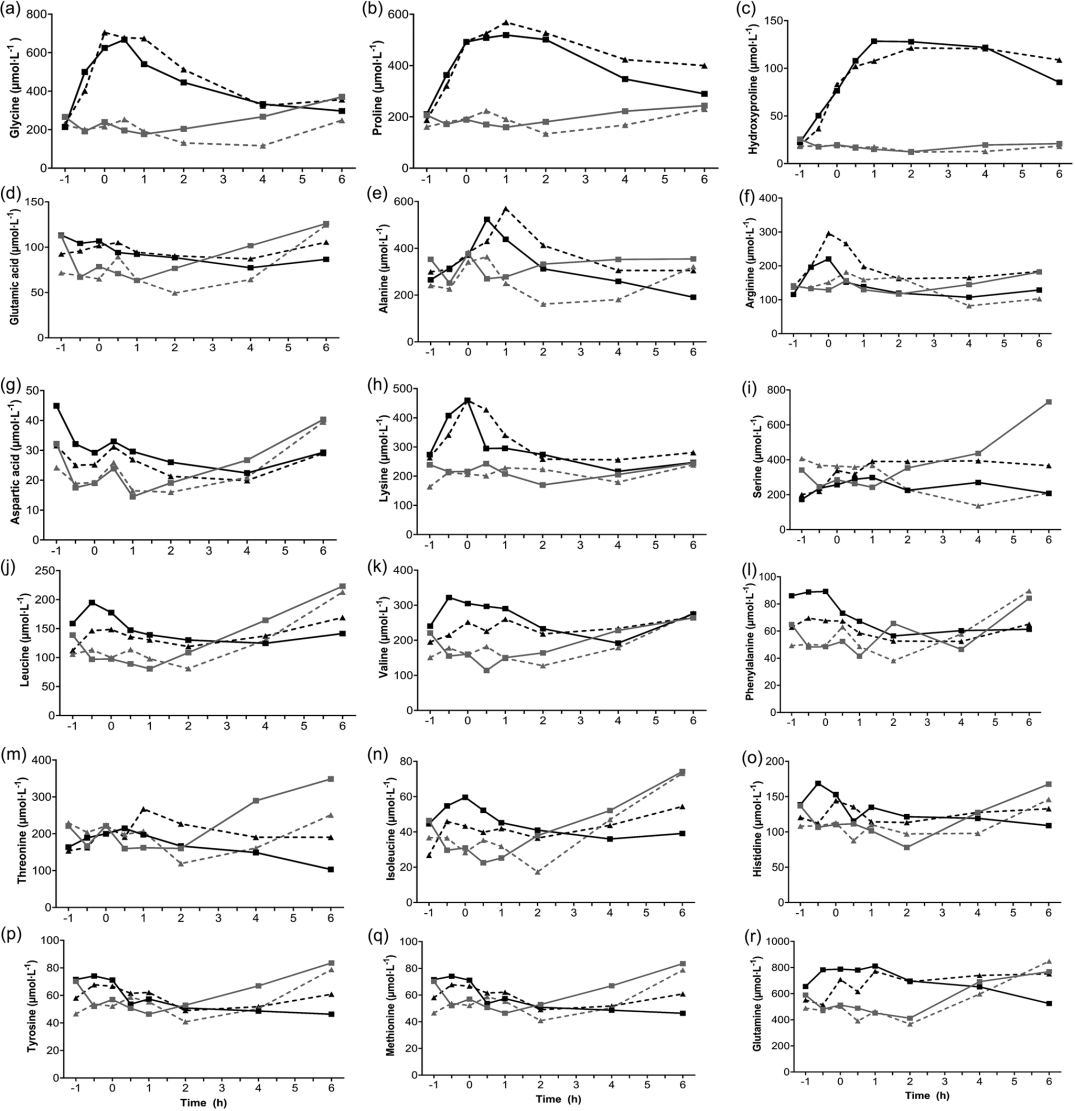
Serum concentrations of 18 collagen amino acids following ingestion of 30 g hydrolysed collagen (HC, black squares) or 0 g HC (grey squares) at the onset of menses; and 30 g HC (black triangle) or 0 g HC (grey triangle) at the end of the late follicular phase.

Serum 17β‐oestradiol concentration was lower at OM than at LF (Figure 2). Serum PINP concentration peaked at +1 h post RE and the PINP concentration × time AUC was greater in the 30 g HC OM (201 μg L^−1^ h) intervention than the 30 g HC LF (144 μg L^−1^ h), 0 g HC OM (151 μg L^−1^ h) and 0 g HC LF (122 μg L^−1^) interventions (Figure 2). The percentage differences in PINP AUCs between interventions are shown in Table 1. Plasma β‐CTX concentration decreased 1.3‐ to 1.5‐fold from rest immediately prior to HC ingestion to 6‐h post RE in all four interventions (Figure 2). The serum concentrations of 18 amino acids that constitute collagen are shown in Figure 3. The concentration × time AUCs of glycine, proline and hydroxyproline in the 30 g HC interventions were 2.2, 2.3 and 6.4 times greater than in the 0 g HC interventions, respectively.

**TABLE 1:** Percentage differences in serum PINP concentration × time AUC between interventions.

Intervention and serum PINP AUC	(2) 30 g HC LF 144 μg L^−1^ h	(3) 0 g HC OM 151 μg L^−1^ h	(4) 30 g HC OM 201 μg L^−1^ h
(1) 0 g HC LF 122 μg L^−1^ h	+18.0%	+23.8%	+64.8%
(2) 30 g HC LF 144 μg L^−1^ h	—	+4.9%	+39.6%
(3) 0 g HC OM 151 μg L^−1^ h	—	—	+33.1%

*Note*: For example, comparing intervention 4 (30 g HC OM) with intervention 1 (0 g HC LF) led to a 64.8% higher serum PINP AUC in intervention 4. Abbreviations: AUC, area under the curve; HC, hydrolysed collagen; LF, late follicular phase; OM, onset of menses.

## 4 DISCUSSION

We aimed to compare collagen turnover following a single bout of high‐intensity resistance exercise (RE) with and without ingestion of 30 g hydrolysed collagen (HC) in a healthy, young, naturally menstruating, female CrossFit athlete at different stages of her menstrual cycle, that is when circulating oestrogen concentration was low at the onset of menses (OM), and when it was high towards the end of the late follicular phase (LF). To our knowledge, this is the first study to investigate the individual and combined effects of HC ingestion prior to RE and different concentrations of circulating oestrogen on collagen turnover exclusively in a female athlete. Our results demonstrated that high circulating oestrogen was associated with a lower whole body collagen synthesis response after RE but ingesting HC at LF appeared to offset this physiological disadvantage. Ingesting HC when oestrogen was low, that is at OM, was even more advantageous regarding the collagen synthesis response to RE.

Peak serum PINP concentrations were 5.6 and 3.3 times greater than baseline values following high‐intensity RE at OM and during LF, respectively. This is in line with previous reports of an acute bout of resistance‐type exercise increasing circulating PINP concentration in young, healthy women, who did not use the oral contraceptive pill (Hansen et al., 2008; Miller et al., 2007). Moreover, we found that the PINP concentration × time AUC in 30 g HC at OM and LF was 1.3‐fold greater than when no HC was ingested. These findings are in line with the study by Lee et al. (2024) in which 30 g HC ingestion with a bout of RE augmented PINP concentration × time AUC greater than 0 g HC ingestion in resistance‐trained young men. It is possible that HC ingestion exerted its effect on collagen synthesis via different mechanisms compared to RE. Firstly, the greater availability of key exogenous amino acids following 30 g HC ingestion (Figure 3) may have simply provided more of the necessary constituents for a RE‐induced increase in collagen synthesis. Secondly, the higher concentration of these amino acids, known to stimulate collagen synthesis independently of RE (Surazynski et al., 2010; Szoka et al., 2017) may have activated key signalling pathways (i.e. Akt/mammalian target of rapamycin complex 1 (mTORC1)) within our athlete's connective tissues that promote collagen synthesis independently of RE. The combination of these two potentially independent mechanisms may explain the greater collagen synthesis response when 30 g HC compared to 0 g HC was ingested at both phases of the menstrual cycle.

In addition, we observed that the PINP × time AUC was higher at OM than at LF, that is whole body collagen synthesis was higher when endogenous oestrogen concentration was low, which contrasted with a reduction in collagen synthesis when oestrogen concentration was high. Specifically, the AUC of 0 g HC at OM (low oestrogen concentration) was 23.8% higher than 0 g HC at LF (high oestrogen concentration) (Table 1). However, ingesting HC prior to RE appears to rescue the serum PINP response when circulating oestrogen is high (30 g HC at LF), but the positive effect of HC ingestion is much greater when circulating oestrogen concentration is low (30 g HC at OM) (Table 1; Figure 2). Although the exact mechanism(s) underpinning these findings cannot be inferred from our study, it is possible that oestrogen directly inhibits collagen synthesis. For example, when porcine anterior cruciate ligament fibroblasts are mechanically loaded and administered with different concentrations of 17β‐oestradiol, an inverse relationship exists between 17β‐oestradiol concentration and type I collagen mRNA expression (Lee et al., 2004). Alternatively, oestrogen may indirectly inhibit collagen synthesis by interacting with insulin‐like growth factor I (IGF‐I). In postmenopausal women, systemic and tendon IGF‐I concentrations were approximately 1.4 and 2.3 times lower in hormone replacement therapy (HRT) users who took 2 mg oral 17β‐oestradiol daily, compared to age‐matched non‐HRT users (Hansen et al., 2009). As mechanical loading upregulates procollagen I α2 and IGF‐1 gene expression in rat plantaris tendon, resulting in augmented tendon mass (Olesen et al., 2006), the lower collagen synthesis observed in our study when circulating oestrogen was high (i.e., at LF) may be due to an inhibitory effect of oestrogen on IGF‐I secretion during muscle contraction.

Contrary to our findings, Miller et al. (2007) reported that serum oestrogen concentration was not associated with serum PINP concentration or tendon collagen synthesis following exercise in healthy young women. However, the two phases of the menstrual cycle when oestrogen was measured, that is early follicular (3 days after OM) and luteal (4 days after ovulation), were probably not the most appropriate for assessing maximal differences in circulating oestrogen concentration, given the overlap in their oestrogen values (70–420 pmol L^−1^ in the early follicular phase and 240–90 pmol L^−1^ in the luteal phase), and the fact that oestrogen peaks just before ovulation, that is at LF, not the luteal phase (Hansen et al., 2008; Miller et al., 2007). Unsurprisingly, the mean circulating oestrogen concentration measured 2–3 days after OM (210 ± 100 pmol L^−1^) in the study by Miller et al. (2007) was similar to our measure at OM (180 ± 13 pmol L^−1^). However, the mean oestrogen concentration measured in the luteal phase (420 ± 200 pmol L^−1^) in the study by Miller et al. (2007) was roughly half the concentration measured in our athlete at LF (891 ± 116 pmol L^−1^), which again is perhaps unsurprising given that oestrogen secretion peaks at LF, not in the luteal phase. However, it should be noted that serum oestrogen concentration varies between women when measured at the same relative point in the menstrual cycle (Miller et al., 2007), so it is not known if we would have arrived at the same conclusions had we recruited a larger cohort of female athletes.

Concerning collagen degradation, plasma β‐CTX concentration was lower at 6 h post‐RE compared to 1 h prior to RE in the current study, regardless of collagen dose or menstrual cycle phase. Qvist et al. (2002) found that human serum CTX‐I concentration was higher in the morning (08.00 h) and started to decrease from 11.00 to 14.00 h independently of exercise. Thus, as β‐CTX at 6 h post‐RE was measured at 15.00 h in the current study, it is possible that circadian rhythm may have affected these results. However, unpublished data from our laboratory suggest the decrease in β‐CTX occurs immediately after RE and remains low for the subsequent 6 h of rest, regardless of HC dose. It is therefore possible that the high‐intensity RE model used in the current study may have inhibited collagen breakdown with immediate effect, lasting for the remainder of each intervention.

A limitation of this case report is the sample size. Given the variability in endogenous oestrogen concentration between and within eumenorrhoeic females (Shultz et al., 2011), it remains to be seen if we would have seen the same pattern of responses with a statistically powered sample size. Therefore, future studies need to investigate the effect of different endogenous oestrogen concentrations with RE and HC ingestion on collagen synthesis with an appropriate sample size of naturally menstruating female athletes. It should also be noted that all four interventions in this case report were performed with the athlete in the fasted state. This was necessary to ensure that any effect on serum PINP or plasma β‐CTX concentration was due to the resistance exercise, HC ingestion and/or oestrogen, and not to the ingestion of other food sources (that may or may not have contained collagen amino acids). For example, β‐CTX concentration is known to be affected by food consumption (Szulc et al., 2017). However, remaining fasted for such a prolonged period of time does not necessarily represent a real‐world environment, and it remains to be seen if we would have replicated our results had we incorporated regular, controlled feeding throughout each intervention.

To conclude, high circulating oestrogen concentration was associated with lower whole body collagen synthesis following RE in a female CrossFit athlete. Ingesting 30 g HC prior to performing RE, however, augmented the collagen synthesis response at both LF and OM, with an overall superior collagen synthesis response at OM. These findings have significant implications for naturally menstruating athletes, as the late follicular phase has been associated with greater joint laxity and higher injury risk and may be linked to the reduction in collagen synthesis we observed during this high oestrogen phase, which may weaken connective tissues, such as tendon, ligament and muscle. Also, a more compliant muscle–tendon unit during the late follicular phase would be expected to reduce the rate of force development, thus decreasing athletic performance. Thus, naturally menstruating female athletes may wish to supplement their training with HC (particularly during the late follicular phase), as it may help protect their connective tissues from injury, whilst maintaining physical performance throughout the menstrual cycle.

## AUTHOR CONTRIBUTIONS

Authors contributed to this manuscript as follows: study conception and design: Robert M. Erskine; data collection: Joonsung Lee; data curation: Joonsung Lee; data analysis: Joonsung Lee, Robert M. Erskine, Jonathan C. Y. Tang, Rachel Dunn, John Dutton; supervision: Robert M. Erskine, David R. Clark, Claire E. Stewart; writing—original draft: Joonsung Lee; writing—review, editing and approval of final draft: Robert M. Erskine, Jonathan C. Y. Tang, Rachel Dunn, John Dutton, William D. Fraser, Kevin Enright, David R. Clark, Claire E. Stewart. All authors have read and approved the manuscript and agree to be accountable for all aspects of the work in ensuring that questions related to the accuracy or integrity of any part of the work are appropriately investigated and resolved. All persons designated as authors qualify for authorship, and all those who qualify for authorship are listed.

## CONFLICT OF INTEREST

The authors declare that the research was conducted in the absence of any commercial or financial relationships that could be construed as a potential conflict of interest.

## FUNDING INFORMATION

None.

## Data Availability

The data that support the findings of this study are available from the corresponding author upon reasonable request.
